# Speed Estimation of Six-Phase Induction Motors, Using the Rotor Slot Harmonics

**DOI:** 10.3390/s22218157

**Published:** 2022-10-25

**Authors:** Khaled Laadjal, Fernando Bento, Hugo R. P. Antunes, Mohamed Sahraoui, Antonio J. Marques Cardoso

**Affiliations:** 1 CISE—Electromechatronic Systems Research Centre, University of Beira Interior, Calçada Fonte do Lameiro, P-62001-001 Covilhã, Portugal; 2 LGEB, Department of Electrical Engineering, University of Mohamed Khider Biskra, Biskra 07000, Algeria

**Keywords:** six-phase induction machine, speed estimation, speed sensorless control, rotor slot harmonics (RSH), Short Time Fourier Transform (STFT)

## Abstract

Multiphase machines have recently been promoted as a viable alternative to traditional three-phase machines. Most experts are looking for strategies to estimate the rotation speed of such complex systems, since speed data are required for high-performance control purposes. Traditionally, electromechanical sensors were used to detect the rotor speed of electric motors. These devices are extremely accurate, but they are also delicate and costly to deploy. New speed estimating algorithms must be created for these situations. This paper looks at how to estimate rotor speed in symmetrical six-phase induction motors (IMs) using a novel strategy for rotor speed estimation based on the Short Time Fourier Transform (STFT) method. The technique is based on tracking the frequencies of the rotor slot harmonics (RSH) seen in most squirrel-cage IM stator currents, thus assuring a broad range of applications. To monitor the RSH, the STFT employs a sliding window to perform the discrete Fourier transform technique, making it more suitable for online use with noisy and nonstationary signals. Experimental tests demonstrate the effectiveness of the suggested approach.

## 1. Introduction

**Figure 1:**
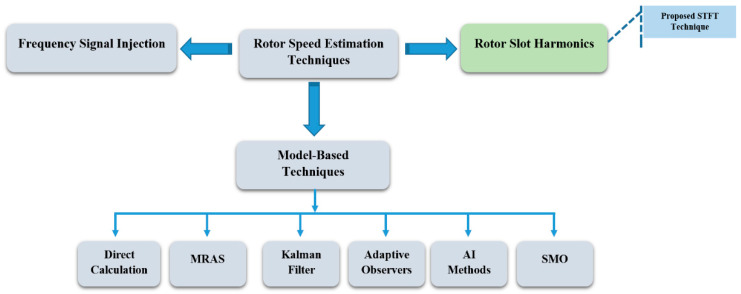
Classification of speed estimation methods for sensorless systems.

Three-phase induction machines (IMs) are extensively used in many industrial applications for their simplicity, robustness, and reliability. Nevertheless, for specific fields where high reliability is required, such as automotive, aerospace, military, and nuclear, it appears that using classical three-phase IMs is no longer suitable, since loss of one or more phases compromises the operation of the whole system. To improve reliability of IMs, increasing the number of phases on the machine and converter sides, in such a way as to obtain a multiphase system, is seen as a promising solution [1,2]. Multiphase drive systems have been widely investigated because of their inherent advantages when compared to three-phase ones, such as (1) reduced current ratings processed by each converter leg, (2) lower torque pulsations, (3) fault tolerance capability, and (4) the ability to eliminate the common mode voltage, amongst others [3,4]. In such complex motor drives, the use of speed sensors brings several concerns because they typically reduce the system reliability, increase overall system costs, and imply extra installation space. Hence, speed sensorless control, implemented through speed estimation schemes, is increasingly demanded in IM drives [5]. In the current literature, there are two types of speed estimation systems, considered in IM drives: non-ideal phenomena-based schemes and model-based schemes. Non-ideal, phenomena-based schemes can either be deployed through signal injection approaches [6,7,8,9] or rotor slot harmonics (RSH) extraction [10,11,12]. Figure 1 below provides a classification of state-of-the-art speed estimation schemes.

Even though signal injection systems operate well across a large speed range and are unaffected by motor parameter changes or uncertainties, they may have undesirable side effects on IM drives, such as noticeable torque ripples and excessive power losses [13,14]. Furthermore, common disadvantages like difficult signal processing and limited flexibility limit their interest for practical implementation. In turn, model-based approaches rely on an analytical representation of the motor to accomplish speed estimation [14,15,16,17,18,19,20,21,22]. The employment of observers or parametric techniques for this purpose is common among model-based approaches. Given the dependence of model-based estimation schemes on motor parameters, unreliable estimation results may be attained in the presence of parameter variations or parameter uncertainty. The literature provides a relevant set of model-based estimation techniques. In practice, model-based estimation schemes are implemented by employing specific observers. Observers require detailed knowledge about the motor settings. Most of those settings change dynamically during the starting process. For IMs, such conditions are more common in motors with star–delta connections or variable rotor resistance. This indicates that IM specifications are required for this sort of speed estimation system. In [19], flux estimation is employed to identify parameters that are independent of the speed of the observer’s output. In [23], model-based solutions are used to obtain accurate estimations in the high-speed range—an operation region where speed estimation is particularly challenging. The performance of the observers, and therefore the speed estimation in steady- and transient-states, is enhanced by speed estimation algorithms that are less susceptible to multi-parameter perturbations [24]. Additionally, within the category of model-based approaches, an improved AFO was described in [14] for speed-sensorless-controlled IM drives, in which the gain matrix was intentionally created to provide high resistance against undesired disturbances. Various observers, such as the adaptive full-order observer (AFO) [14,15,16], adaptive reduced-order observer (ARO) [25], extended Kalman filter (EKF) [26], and sliding mode observer (SMO) [27,28], are used to create model-based estimation systems. The use of an optimised constant-rate reaching law was proposed in [20] to increase estimation performance. A review of AFO-based systems for IM drives, based on stator and rotor resistance detection, was first presented in [16]. After that, an effort was made to enhance the AFO scheme through simultaneous calculation of stator and rotor resistances, along with stability analysis for steady-state operation. A novel speed-sensor-free approach for IM drives, based on the ARO, is described in [25]. The flux estimation was removed from this technique, simplifying the overall system and parameter adjustment. The stability study of the suggested speed estimator was also presented in accordance with the singular perturbation theory [25]. In [26], a complete performance comparison between optimised EKFs, employing different fitness functions, was provided. Instead of using complicated covariance matrices, the differential evolution algorithm and the multi-objective differential evolution method were used. This allows for precise speed estimation and a reduction in computation load.

An improved full-order sliding-mode observer for rotor position and speed estimation of SPMSM has been proposed in [27]. This solves the problem of bounded disturbances causing instability. In [28], a second-order SMO approach for linear IM drives was developed. This method allowed for more precise speed estimations and minimal oscillation. To address the issue of dynamic stator resistance, a parallel stator resistance on-line identification scheme was used. Model reference adaptive system (MRAS) methods are appealing since they are simple and require little computation effort. In [21], a review of MRAS-type systems for motor drives was conducted, which provides a comprehensive overview of numerous MRAS-based systems and their stability prospects. Advanced control methods, such as the particle swarm optimization approach [29], adaptive full-order observer [30], fuzzy control method [31], genetic algorithm [32], artificial neural network (ANN) method [33,34], and nonlinear control method [35], can also be considered to estimate speed. The majority of modern control-based systems have demonstrated excellent accuracy in speed estimation. Nonetheless, serious concerns prevail for both non-ideal phenomena-based and model-based speed estimation schemes, such as high computation burden and high complexity. In addition, the adoption of state-of-the-art speed estimation schemes for the most recent motor drives—multiphase machines, permanent magnet machines, synchronous reluctance machines—remains extremely challenging. In particular, the significant number of degrees of freedom of multiphase machines makes the implementation of model-based schemes very demanding.

Finally, rotor speed estimation can be accomplished through RSH extraction, which takes advantage of machine saliencies. The saliencies may be caused by rotor structural modifications or magnetic saturation. In the case of squirrel-cage IMs, the saliencies are attributable to the rotor slotting [36,37]. Even though RSH extraction is commonly considered for defect identification in IMs [38], several investigations have also employed RSH for speed estimation purposes since their frequencies are fundamentally associated with the motor speed [36,39,40]. The effectiveness of RSH speed measuring methods is highly dependent on the approach considered for spectral analysis. Particular attention should be paid to the effectiveness of dealing with nonstationary signals. Several strategies have been presented in the literature in this regard. The earliest published study on this area focused on RSH extraction using fast Fourier transform (FFT)-based algorithms [41]. The FFT is a nonparametric method for converting a periodic, discrete signal from the time domain to the frequency domain using a finite number of data samples. Although the FFT is a simple and quick approach, it has a number of major flaws that limit its applicability and effectiveness. To attain good frequency resolution, the FFT requires a long acquisition period. In addition, the FFT is only useful for stationary harmonics. The rotor slot spectral components, on the other hand, are time-varying, and steady-state conditions can be lost with time [42]. Recently, various enhancements have been made to address the shortcomings of FFT-based approaches. For example, the dichotomous search method was paired with FFT analysis to improve frequency extraction accuracy. In a wound rotor IM, this approach was used to estimate speed without using sensors [43]. In [44], the rotor speed was calculated using the chirp Z transform (CZT). The RSH of a squirrel single-cage IM was calculated using this method. Thanks to the narrow observation window and superior spectral resolution over FFT-based approaches, CZT has been found to deliver improved accuracy. In [45], the maximum covariance approach for frequency tracking (MCFT) was effectively employed to estimate and monitor the fundamental component and the RSH, even under time-varying settings. This approach looks at the cross-correlation function between the noisy input signal and the reference signal and delivers high frequency resolution, regardless of the sampling frequency or time acquisition. Notwithstanding the MCFT merits, this approach takes a significant amount of computation time when a broad frequency bandwidth and very high frequency resolution are required, limiting its capability to perform on-line speed estimation. The traditional multiple signal classification (MUSIC) approach is also a powerful instrument that has been widely utilized for frequency detection [46,47]. Although MUSIC may extract high-frequency resolution from a short data record signal buried in noise, it has the disadvantage of taking a long time to compute as the autocorrelation matrix grows. For calculating the instantaneous rotational speed, the harmonic decomposition (HARD) approach was devised. The HARD technique is characteristic of eigenvalue-based harmonic identification methods, which have higher precision and accuracy in detecting harmonic components, even in noisy situations. In [37], another helpful technique based on frequency demodulation was proposed. Based on the monitoring of a single harmonic component of the rotor on the spectrogram of the stator currents, a self-sensing approach for IM speed calculation during the initial acceleration transient was provided in [48]. This methodology implies the measurement of all three stator currents and the computation of the α-β components, making it challenging for online implementation on 6PIM drives. In [49,50], another technique suitable for rotor speed estimation is presented. It can correctly estimate the rotor speed from the RSH frequency, over the entire IM speed range. Prony’s analysis (PA) is a compelling approach to estimating the harmonic content of a signal. PA is a high-resolution spectral analysis approach based on the original work of Gaspard de Prony, a French mathematician. Using this approach, a sampled waveform is approximated as a linear combination of complex conjugate exponentials. From a brief data logging signal, the Short Time Least Square Prony’s (STLSP) can correctly estimate all harmonic attributes: frequency, amplitude, phase, and damping factor. The features of PA solve the shortcomings of the discrete Fourier transform (DFT) and other state-of-the-art speed estimation approaches. Nonetheless, the STLSP technique reveals weaknesses for speed estimation applications since the filtering, DC component removal, and down sampling procedures, especially for high-order systems like multiphase motor drives, require a long and complicated processing time.

To overcome the drawbacks identified in the literature, a practical and efficient approach to estimating and tracking RSH frequencies is provided, based on a modified version of the DFT method. Only one stator current is measured in a non-invasive manner. The measured stator current is divided into short overlapping time windows, and each window is evaluated using the Short Time Fourier Transform (STFT) method, which allows evaluation of both the stationary and nonstationary parts of the problem. The suggested method generates a linear time representation of RSH frequencies with high frequency accuracy and variable time resolution. Thanks to such features, accurate speed estimation and low computational effort are attainable regardless of the motor drive complexity, making the proposed approach not only suitable for all motor drive technologies but particularly compelling for multiphase IM drives.

## 2. The Theory of the Proposed Technique

To track the harmonic components considered useful to estimate speed, it is preferable to remove both the fundamental component of the power grid and the harmonics induced by the stator currents. It is a fact that the amplitudes of the electrical grid’s harmonic components prevail over the amplitudes of the rotor’s harmonics. In addition, such harmonics are often observed within a small spectral window. As a result, inaccuracies in the speed estimation technique might occur. Therefore, filtering the signal to be studied is a good practice. Comb filters and multi-rate filters are the most common frequency-domain filters for removing harmonic interference [23].

**Figure 2:**
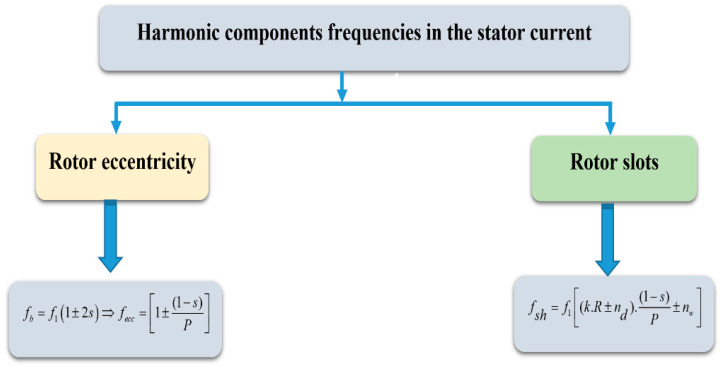
Harmonic components’ frequencies in the stator currents.

If supplied with balanced voltages, IMs show sinusoidal rotor and stator currents and their magnetic fields rotate at a frequency of *f*_1_ in the stator reference frame or *sf*_1_ in the rotor reference frame, where the supply voltage frequency is *f*_1_, and the IM slip is *s*. Even in a healthy IM, however, the existence of harmonic components in both rotor and stator currents is determined by specific construction characteristics or defects. Figure 2 describes the expressions that identify the frequencies of the harmonic components generated in the stator currents by different constructive characteristics of IMs or those generated by faults, namely eccentricity. Referring to the expressions in Figure 2, *k* denotes a positive integer (0,1,2,3,4,5,…), *R* is the number of rotor slots, *n_d_* is an integer (0,±1,±2,±3,±4,±5,…), *P* is the number of pole pairs, and *n_w_* is the harmonic order (±1,±3,±5,±7,±9,…). The uneven distribution of conductors in the rotor slots creates steps in the rotor magneto-motive force (MMF) that induce air-gap flux modulation with a speed-dependent frequency in squirrel-cage IMs, which are the most common motors in the industry. Variations in the air-gap flux are reflected in the stator currents because the air-gap flux is linked to the stator windings. The spectral components that arise are commonly referred to as RSHs in the literature. Because their frequencies are not multiple integers of the fundamental harmonic component, RSHs are formally classed as interharmonics.

All harmonics that may be measured in the general case have frequencies given by the well-known generalised form [51]:

(1) $$f_{h}=[(k.N_{b}\pm n_{d})f_{r}\pm \nu f_{s}]$$

where *f_r_* is the mechanical rotor speed in Hz and is given by $f_{r}=\frac{\left(1-s\right)}{P}f_{s}$, s is the motor slip, *ν* denotes the time harmonic order, *f_s_* the fundamental frequency, *N_b_* the number of rotor slots, and *n_d_* denotes the eccentricity order. The general formulation of the RSH frequencies is determined in the situation of a healthy motor (*n_d_* = 0). Still, harmonics linked to mechanical faults, such as air-gap eccentricity, are also covered by this equation. IMs are often powered by non-sinusoidal voltages for driving purposes. As a result, each supply component creates a pair of RSHs in the line current spectrum. The dominant RSHs are those produced by the basic component (*ν* = 1), and they generally have a higher magnitude. These dominant RSHs are commonly called principal slot harmonics (PSHs), and their magnitude is always greater than the spectral noise. The PSH frequencies are given by the following expression:

(2) $$f_{PSH1,2}=\left(N_{b}.f_{r}\pm f_{s}\right)$$

It is sufficient to examine the frequency of only one PSH to accurately determine the motor speed. As a result, the motor speed can be expressed in *Hz* as:

(3) $$fr=\frac{\left(f_{PSH1}-f_{s}\right)}{N_{b}}\;\Rightarrow \;n_{r}=60\frac{\left(f_{PSH1}-f_{s}\right)}{N_{b}}$$

**Figure 3:**
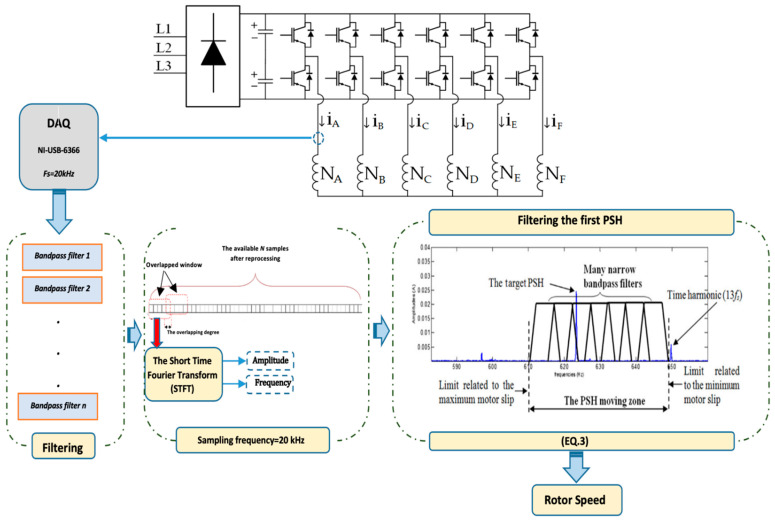
Description of the proposed method.

On the other hand, fluctuations in the air-gap permeance occur when eccentricity or any other mechanical defect ($n_{d}\neq 0$) occurs. Such fluctuations interact with existing time and space MMF harmonics, producing spectral components around the existing PSHs. The amplitudes of the sidebands surrounding the PSH do not match those of the PSHs themselves—this is due to the fact that PSHs are the root cause of these sidebands. Therefore, the effective estimation of the speed remains feasible even in the presence of mechanical faults. The STFT technique, which is based on applying the DFT algorithm to a short-time sliding window, is used in this approach. This method is straightforward, quick, and capable of properly determining and tracking the frequency and amplitudes of the desired harmonics. This enables the nonstationary element of the problem to be considered. The STFT method is well suited for finding the first PSH frequency since this method was originally developed to estimate and track any spectral component. Only preprocessing of the acquired signal is required to succeed in estimating the PSH frequency through the STFT method. A general description diagram showing the STFT method is illustrated in Figure 3. In this paper, filtering the stator current has two main objectives: attenuation of noise and separation of the first PSH from undesired spectral components. To accomplish such objectives, a narrow bandpass filter is designed and employed to process the current signal after acquisition (Figure 3). The parameters of this filter are specified to allow passing only the first PSH. As can be understood, the STFT requires analysing the signal in a narrow band frequency, as opposed to the DFT method, which analyses the entire signal spectrum. Any sort of signal processing, including time-domain, frequency-domain, or time-frequency analysis, can be used to derive a signal-based speed estimation. As shown in Figure 3, the suggested technique is implemented in real-time, using basic phase stator current measurements [26,27,51]. The STFT approach is then used to estimate and track the amplitude and phase angle of the PSH component. The following are the stages of implementation of the algorithm:

➢ Acquisition of one-phase current $\left(I_{a}\right)$;

➢ Frequencies determination: The DFT transforms a finite discrete-time signal into a finite or discrete number of frequencies. For a finite sequence of length *n* {*x*(*n*) 0 ≤ *n* ≤ *N*−1}, the DFT is given by (4), but the angular frequency is continuous. This constraint is solved by discretising the frequency; the resulting DFT is given by (5). Equation (5) has a finite sequence length and is repeated periodically {*x*(*n*) = 0, *N* ≤ *n* ≤ *L*−1}, and is thus obtained from (4) as follows [51]:

(4) $$X\left(e^{jw}\right)=\overset{N-1}{\underset{n=0}{\sum }}x\left(n\right).e^{-jwn}$$

(5) X(k)=X(ejw)w=2πkL=∑L−1n=0x(n).e−j2πnk/l

➢ The inverse DFT (6) is obtained from (5) as:

(6) $$x\left(n\right)=\overset{L-1}{\underset{k=0}{\sum }}X\left(k\right).e^{j2\pi nk/l};$$

➢ Estimation of the magnitude and frequency of the PSH related to the phase current: A high-resolution spectral analysis, based on the STFT technique, is used in this phase of the strategy. The STFT is based on a short-time sliding window and the DFT method. This approach can reliably estimate and monitor all harmonic properties (frequency and amplitude) from a short data record signal, allowing the non-stationary part of the problem to be considered [51].

When employing single harmonics, more than one curve may match the two requirements indicated above at the same time. In such a situation, the curves are quite likely to coincide or be very near to each other, allowing one to choose one without making too many estimation errors. If this is not the case, the comb filter may need to be tweaked to eliminate grid harmonic effects, or the measured signal may be excessively noisy.

## 3. Experimental Results

**Figure 4:**
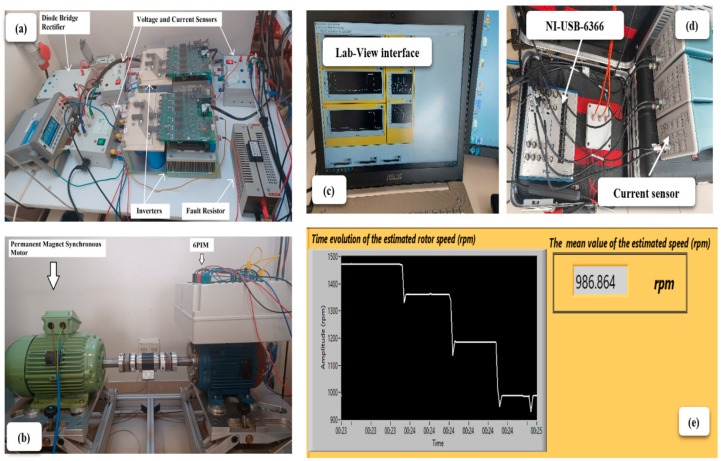
General view of the experimental test bench. (**a**) The used inverters; (**b**) The used 6IMs; (**c**) Lab-View interface; (**d**) DAQ (NI-USB-6366) and current sensor; (**e**) Estimated rotor speed.

The experimental setup shown in Figure 4 was created to test the performance of the speed estimation methodologies on a six-phase IM (6PIM). The 6PIM considered in the experiments, whose characteristics are listed in Table 1, was designed using a dual-speed IM with Dahlander windings (1.5 kW–1460 rpm and 6 kW–2930 rpm). To preserve the original rated current, the same conductor gauge and number of conductors per slot were used. A permanent magnet synchronous generator is mechanically coupled to the motor, providing mechanical load to the 6PIM. The power converter, supplied through a three-phase auto-transformer, is made up of a diode bridge rectifier and two Powerex POW-R-PAK three-phase voltage source inverters, controlled by a dSPACE DS1103.

A voltage/frequency scalar control was created in Matlab/Simulink and integrated into the dSPACE controller. A sinusoidal pulse width modulation control strategy with a triangular carrier wave at a frequency of 5 kHz is considered for the scalar control. A sample time of 20 μs was used for both the control and data acquisition.

The load conditions were controlled through the permanent magnet synchronous generator, and the stator currents were measured with Hall-effect clamp probes. A MAGTROL TMB-310/411 torque sensor is used to determine both the real motor speed and load torque (Figure 4b). The data acquisition board NI USB-6366 (Figure 4d) was used to complete the acquisition operation. The instrumentation amplifier buffers and conditions the incoming analogue signal on each channel before sampling it using a 16-bit analog-to-digital converter (ADC). Independent track-and-hold amplifiers on each channel allow simultaneous sampling of all channels. The captured data are sampled at 20 kHz and sent via USB connection to the personal computer (Figure 4c).

**Figure 5:**
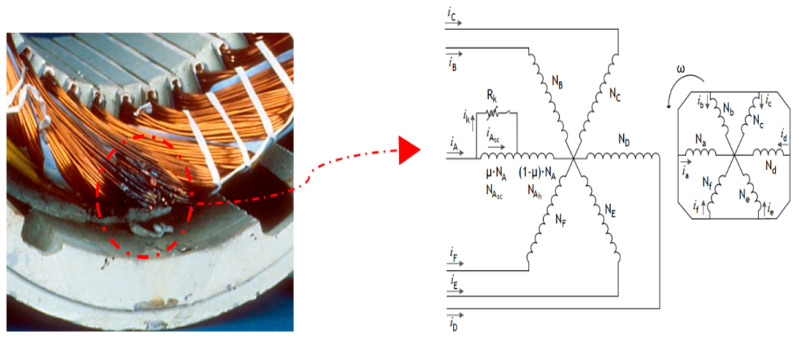
ITSC fault on a stator winding of a 6PIM.

**Figure 6:**
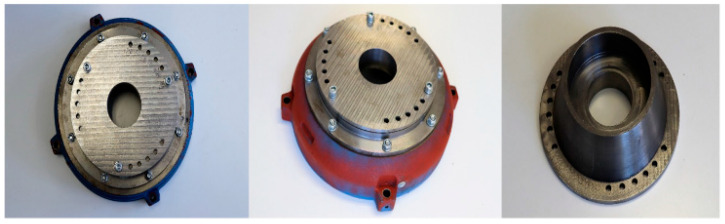
Eccentricity fault mechanism on a 6PIM.

For experimental purposes, the 6PIM is assumed to be working with a variable load torque. It consists of a mean value of 80% of the rated load torque and an arbitrary variation around that value. In addition, rapid changes in speed are considered under both healthy and faulty states—inter-turn short circuit (ITSC) and eccentricity faults. Both ITSC faults and eccentricity faults pose challenges to the effective speed estimation. For that reason, the proposed speed estimation approach is also tested in the presence of such fault conditions. An ITSC fault causes a proportional decrease in the resistance of the injured phase, as well as a slight increase in that phase current and high-amplitude current flowing through the shorting branch, resulting in a dangerous increase in temperature, most likely causing insulation and winding destruction. Figure 5 illustrates the effects of an ITSC fault in the motor stator’s windings. In turn, the eccentricity mechanism adopted in the study is shown in Figure 6.

The STFT technique was initially created using MATLAB code, in order to apply the suggested speed estimation method on-line. Using the MATLAB Script Node, the produced code was then imported into the LabVIEW program. The LabVIEW palettes were used to conduct the additional parts of the suggested approach, namely low-pass and band-pass filtering. As previously stated, the PSH frequency is determined by the motor load situation. As a result, four narrow bandpass filters covering the whole frequency range were created, restricted by the lowest and maximum motor slip. The PSH frequency, on the other hand, is dependent on the supply frequency. As a result, the bandpass filter settings must be adjusted based on the source fundamental frequency.

**Figure 7:**
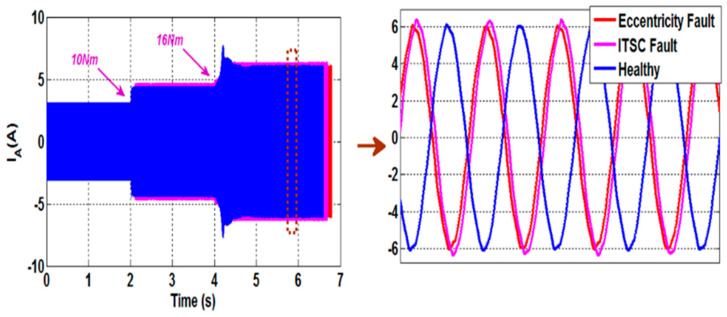
Phase A stator current with the presence of sudden load transients in a healthy motor, a faulty motor with an ITSC fault, and a faulty motor with an eccentricity fault.

**Figure 8:**
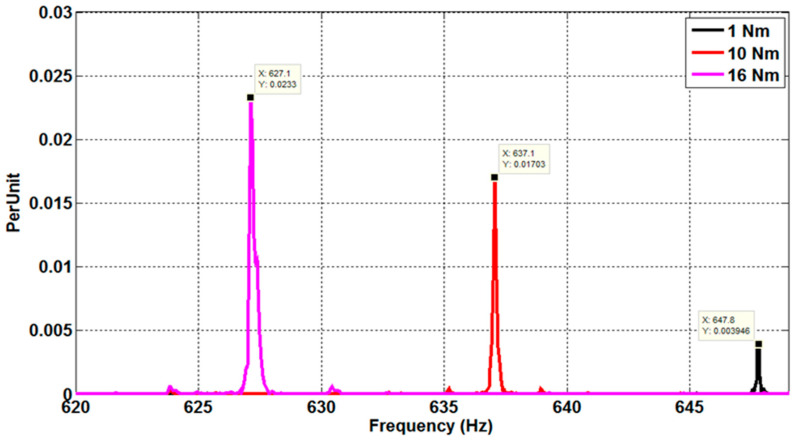
Stator current spectra for different load torque conditions, for a 6PIM with four poles and 28 rotor bars.

The stator currents of a 6PIM with four poles and 28 rotor bars, assessed under healthy conditions, ITSC fault, and eccentricity fault, are shown in Figure 7. The resulting current spectra, for various load torque circumstances, are shown in Figure 8. Referring to Figure 8, it is observed that the motor load has a substantial impact on the PSH frequencies. The PSH frequencies, in reality, travel in a frequency range defined by the lowest and maximum motor slip. As a result, the PSH frequency estimation technique has two main requirements: strong frequency resolution and a limited observation window. These constraints are incompatible with several signal-processing methods, such as the conventional DFT. Indeed, a short observation window provides excellent time resolution but poor frequency resolution, whereas a long window provides excellent frequency resolution and good time resolution. Furthermore, due to supply frequency and load torque fluctuations in variable speed drive applications, the stator currents cannot be regarded as stationary signals. As a result, a signal processing approach suitable to determine the PSH frequencies with fewer sampled points while maintaining spectral resolution is required. The STFT approach successfully accomplishes such goals.

**Figure 9:**
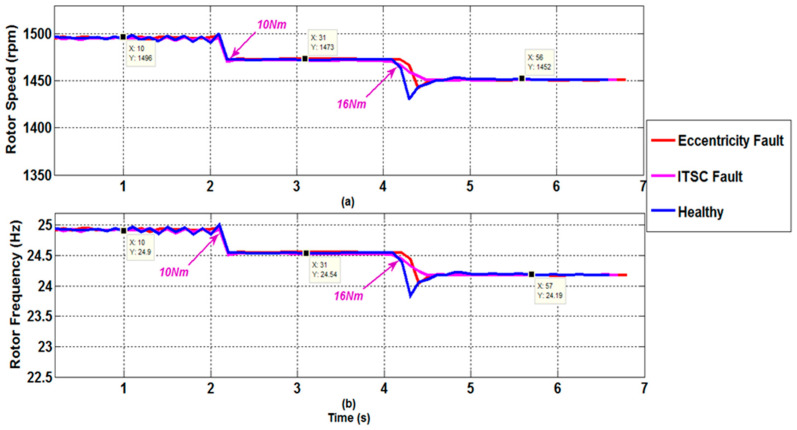
Rotor speed and frequency estimation in the presence of sudden load transients, observed for a healthy motor, a faulty motor with an ITSC fault, and a faulty motor with an eccentricity fault. (**a**) Rotor speed; (**b**) Rotor frequency.

The STFT approach has a number of advantages in nonstationary speed situations. To demonstrate this, operation under sudden load variation was tested. When the motor was running under a load of 1 Nm, the stator current acquisition was initiated. The load was increased to 10 Nm after a few seconds, and subsequently to 16 Nm. The motor slip will naturally increase, and the first PSH will approach the thirteenth time harmonic. To display the estimated rotor speed, a virtual instrument was created using the LabVIEW graphical interface. The findings are shown in Figure 9. At the beginning of the test, the predicted rotor speed was 1496 r/min. When the load was adjusted to 10 Nm, the estimated speed saw a brief shift before settling at 1473 r/min. When the load is further increased to 16 Nm, the projected speed is decreased to 1452 r/min. For heavy and medium loads, the resulting estimation inaccuracy is roughly zero percent in comparison with the observed speed. The estimation error (which is nil) grows in low-load conditions because the target PSH approaches the thirteenth time harmonic. Similar estimation errors are observed in the presence of mechanical or electrical faults—in this case 65% of eccentricity and 23 shorted turns. All these findings indicate that the suggested speed estimator is well suited for condition monitoring applications, particularly non-invasive defect detection. This is because the estimator’s technique is based on examining the stator current of only one phase, which is easily obtained. Furthermore, the STFT approach, which is the heart of the proposed algorithm, can deal with noisy and nonstationary data and is unaffected by motor parameter variations.

**Figure 10:**
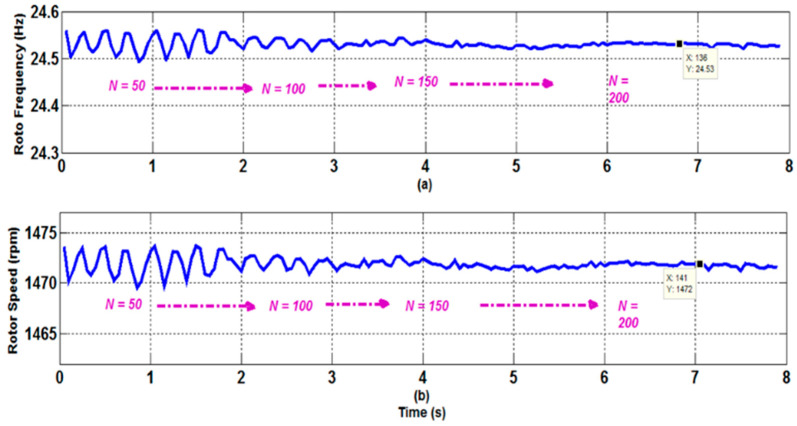
Rotor speed and frequency estimation for a 6PIM as a function of the number of data samples. (**a**) Rotor speed; (**b**) Rotor frequency.

The cost of implementation of the approach is crucial since it involves the inversion of large-size matrices and operations with high-order polynomials. The difficulty of the problem is directly proportional to the number of handled data samples N. In this regard, experiments were carried out with various N (number of treated samples) values to demonstrate the efficacy of the suggested approach, even at 10 kHz. Figure 10 shows the estimated rotor frequency and speed with different values of N. Figure 10 confirms that the suggested methodology provides stable and correct rotor speed estimations, even with a low number of data samples. Furthermore, the suggested STFT approach can handle noisy and nonstationary data while remaining unaffected by changes in system parameters. Even with a longer signal record, the DFT approach, the root of numerous techniques addressed in the literature, only offers information on the amplitudes and frequencies of the essential harmonics. Furthermore, DFT is unable to monitor harmonic features in any way. The STFT approach yields data that are consistent with tach generator readings. Due to measurement noise and harmonic pollution introduced into the system by the switching nature of the inverter, there are minor oscillations in the estimated speed. Despite these challenges, the findings show that the suggested approach for calculating and tracking the rotor speed of a 6PIM powered by non-sinusoidal voltages is successful.

**Figure 11:**
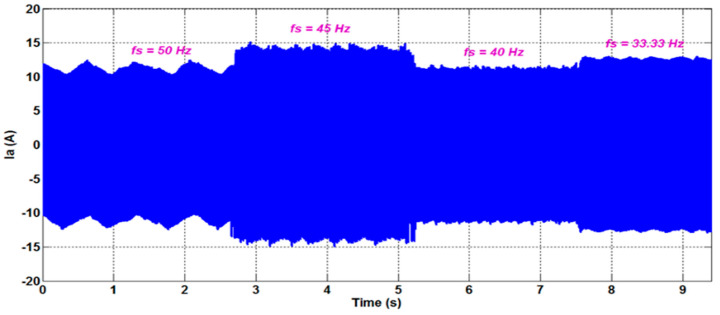
Phase A stator current, measured in the time domain, for a 6PIM operating at a load torque of 10 Nm. The supply current fundamental frequency is progressively adjusted.

**Figure 12:**
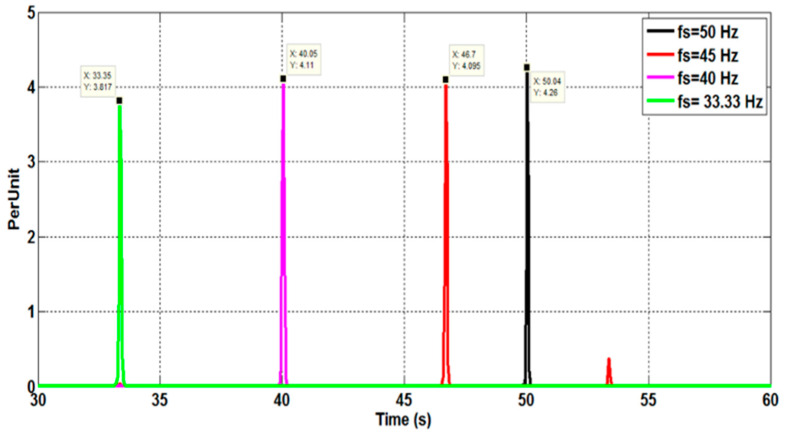
Phase A stator current spectra for a 6PIM operating at a load torque of 10 Nm. The supply current fundamental frequency is progressively adjusted.

**Figure 13:**
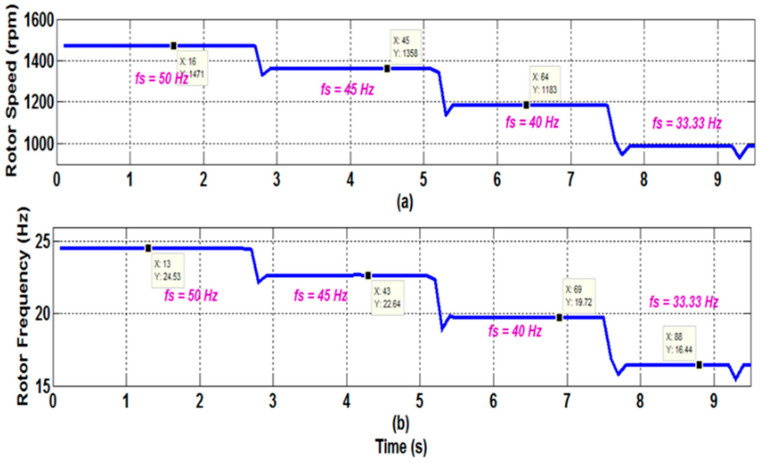
Estimated rotor speed and frequency for a 6PIM operating at a load torque of 10 Nm. The supply current fundamental frequency is progressively adjusted. (**a**) Rotor speed; (**b**) Rotor frequency.

Figure 11 depicts the phase currents measured for various supply frequencies (50, 45, 40, and 33.33 Hz). The purpose of these experimental experiments is to demonstrate the effectiveness of the suggested speed estimation method in variable-speed inverter-fed motor drives. Due to the high amount of harmonic and inter-harmonic pollution in the line current spectra, estimating the PSH frequencies becomes more challenging in this case (Figure 12). Figure 13 illustrates the estimated rotor speed and frequency when sudden variations in the supply frequency are introduced. The results confirm the effectiveness of the proposed techniques to track the PSH and, consequently, to monitor the rotor speed and frequency with high resolution and accuracy (estimation error is almost nil) within a broad speed range.

Estimation accuracy has great importance for any quantitative research project. It shows how consistently the STFT technique can estimate the rotor frequency and speed. In this study, estimation accuracy is evaluated by taking into consideration variations in the motor operation conditions (load, supply frequency, and presence of ITSC faults or eccentricity faults). All tests were performed experimentally and repeated several times to confirm the repeatability of the results. Additionally, online implementation of the proposed speed estimator was performed to verify its effectiveness for practical applications. For the operation conditions evaluated in the study, the estimation error does not exceed 0.013% (Table 2).

## 4. Conclusions

This research introduces a novel rotor speed estimation approach, aimed at 6PIM but equally feasible for other variable-speed motor drives. To determine the motor speed, the method devised for this purpose evaluates the first PSH frequency. The challenge to be addressed consists of figuring out how to precisely identify this harmonic under various operating circumstances with the potential to impact the success of the speed estimation—namely speed variation and faults. On that basis, an online speed estimation tool is presented in this article. The proposed strategy, based on the STFT methodology, is capable of estimating and tracking the amplitude and frequency of any spectral component, making it ideally suited for this application. In addition to the high estimation accuracy, the proposed approach reveals advantages with regard to complexity of implementation, as it only implies the acquisition of one motor phase current.

Compared to the existing methods, the main advantages of the proposed approach are as follows:

(1) Independence from motor parameters, rotor type, or perturbations with the potential to interfere in the estimation procedure—fault-related harmonic components, for instance;
(2) Enhanced performance and reliability for speed estimation of IMs;
(3) Ease of implementation;
(4) Estimation accuracy is higher than 99.987%.

## Figures and Tables

**Table 1:** 6PIM parameters.

Stator phase resistance (*R_S_*)	1.87 Ω
Rotor phase resistance (*R_r_*)	2.98 Ω
Stator per-phase self-inductance (*L_A_*, *L_B_*, *L_C_, L_D_, L_E_, L_F_*)	62 mH
Rotor per-phase self-inductance (*L_a_*, *L_b_*, *L_c_, L_d_, L_e_, L_f_*)	62 mH
Stator per-phase leakage inductance (*L*^σ*s*^)	14.8 mH
Rotor leakage inductance (*L*^σ*r*^)	14.8 mH
Mutual inductance between stator phases (*M_AB_*, *M_AF_*, *M_BC_*, *M_CD_, M_DE_, M_EF_*) and rotor phases (*M_ab_*, *M_af_*, *M_bc_*, *M_cd_, M_de_, M_ef_*)	31 mH
Mutual inductance between stator phases (*M_AC_*, *M_AE_*, *M_BD_*_,_ *M_BF_*, *M_CA_, M_CE_*) and rotor phases (*M_ac_*, *M_ae_*, *M_bd_*, *M_bf_*, *M_ca_, M_ce_*)	−31 mH
Mutual inductance between stator phases (*M_AD_*, *M_BE_, M_CF_*) and rotor phases (*M_ad_*, *M_be_, M_cf_*)	−62 mH
Maximum value of the mutual inductance between stator and rotor phase (*M_SR_*)	62 mH
Moment of inertia (*J*)	0.02 N·m^2^
Torque for losses and friction (*T_av_*)	0.23 N·m

**Table 2:** Rotor speed and frequency estimates in the presence of load and frequency variations.

	*Load Variation (1–16 Nm) (fs = 50 Hz)*											
	***1 Nm***			***10 Nm***					***16 Nm***			
	Frequency (*Hz*)	Speed (*rpm*)		Frequency (*Hz*)		Speed (*rpm*)			Frequency (*Hz*)		Speed (*rpm*)	
Measured Value	24.9	1496		24.54		1473			24.19		1452	
Estimated Value	24.9	1496		24.54		1473			24.19		1452	
Error (%)	0	0		0		0			0		0	
	***Supply frequency variation (10 Nm)***											
	***fs = 50 Hz***			***fs = 45 Hz***			***fs = 40 Hz***			***fs = 33.33 Hz***		
	Frequency (*Hz*)	Speed (*rpm*)		Frequency (*Hz*)	Speed (*rpm*)		Frequency (*Hz*)	Speed (*rpm*)		Frequency (*Hz*)		Speed (*rpm*)
Measured Value	24.53	1471		22.64	1358		19.72	1183		16.44		986.66
Estimated Value	24.55	1472.2		22.67	1358.4		19.75	1183.3		16.48		999.65
Error (%)	0.001	1.2 × 10^−4^		0.0016	2.9 × 10^−4^		0.0019	3.38 × 10^−4^		0.002		0.013

## Data Availability

The study did not report any data.
